# Developmental Pathways of Immature CD11c^+^ Myeloid Dendritic Cells (mDCs) for Bona Fide Osteoclastogenesis Revisited: A Narrative Review

**DOI:** 10.3390/ijms27010480

**Published:** 2026-01-02

**Authors:** Yen Chun G. Liu, Chen-Yi Liang, Andy Yen-Tung Teng

**Affiliations:** 1 Lab. of Adv. Dental Medicine & Hygiene vs. Overall Health, Department of Dental Hygiene, School of Oral Health and Nursing & School of Dentistry, Kanagawa Dental University, Yokosuka 238-8580, Kanagawa, Japan; graceyc@gmail.com; 2 School of Dentistry & Department of Oral Hygiene, College of Dental Medicine, Kaohsiung Medical University, Kaohsiung 80708, Taiwan; 3 For the Elder and Young’s Center of Oral Medicine Investigation (FEYCOMI), 13th F, No.149, Yu-Guo St., Kaohsiung 804506, Taiwan; l10436@mail.cnu.edu.tw; 4 Department of Cosmeceutical and Biotech Industry, Chia Nan University of Pharmacy and Science, Tainan City 717301, Taiwan; 5 The Eastman Institute for Oral Health, School of Medicine & Dentistry, University of Rochester, Rochester, NY 14620, USA

**Keywords:** osteoclast (OC) vs. myeloid OC precursor/mOCp, monocyte/macrophage (Mo/Mϕ) vs. myeloid dendritic cells (mDCs), CD11c^+^ myeloid dendritic cell-derived OC precursor (CD11c^+^ mDDOCp), TRAF6 vs. osteoclastogenic signaling, boneresorption/loss vs. osteoclastogenesis, cytokine crosstalk

## Abstract

Recent studies support that hematopoietic stem cell (HSC)-derived myeloid dendritic cells, monocytes/macrophages (Mo/Mϕ), and osteoclast precursors (OCps) share common progenitor(s) during development. This occurs mainly through receptor activator of NF-κB ligand (RANKL) signaling via its cytoplasmic adaptor protein complex (TRAF6) to subsequent osteoclastogenesis for bone loss and/or remodeling. Presently, mounting new evidence suggests that erythro-myeloid progenitor (EMP)-derived macrophages (Mϕ) and HSC-derived monocytes (Mo) produce embryonic, fetal, and postnatal OCp pools (i.e., primitive OCp), pinpointing a complex network of multiple OCp developmental origins. However, their ontogenic developments, lineage interactions, and contributions to the alternative osteoclastogenesis—in contrast to overall bone remodeling or loss—remain elusive. Interestingly, studies have also elucidated the contributions of immature CD11c^+^ myeloid DC-like OCps to osteoclastogenesis, with or without the classical so-called Mo/Mϕ-derived OCp subsets, and described that CD11c^+^ myeloid DCs (mDCs) develop into functionally active OCs; meanwhile, the cytokine TGF-β mediates a stepwise regulation of de novo immature mDCs/OCps through distinct crosstalk(s) with IL-17, an unrecognized interaction featuring TRAF6^(−/−)^CD11c^+^ mDDOCps that coexist and proficiently colocalize in the local environment to drive a bona fide route for alternative osteoclastogenesis in vivo. Collectively, new findings—critically hinged on progenitor osteoclastogenic pathways (primitive OCps, mDCs/OCps, osteomorphs, etc.) and involving classical and/or alternative routes to inflammation-induced bone loss—are discussed via the illustrated schemes. This review highlights plausible ontogenic vs. principal or alternative developmental paths and their consequential downstream effects.

## 1. Introduction

Bone and its matrices constitute a highly dynamic organ in our body’s skeleton, whose stromal and marrow spaces are filled with multi-lineage cell types interacting progressively or simultaneously at different stages, rather than a discrete hierarchy of processes, which include bone cells (osteoblasts (OBs), osteoclasts (OCs), etc.), both progenitors and mature ones, in or around the bone formation and remodeling units, immunocytes (dendritic cells (DCs), granulocytes (neutrophils, monocytes/macrophages (Mo/Mϕ), etc.), T cells, B cells, etc.), mesenchymal cells, and matrix constituents. These are intricately involved in triggering and fine-tuning immunity, leading to downstream sequelae at the osteo-immune and/or mucosal–mesenchyme interface throughout the lifetime [1,2,3,4]. It is known that ~10% of an adult’s total skeleton is remodeled yearly through a continuous processes involving unique coupling and opposing interactions among bone cells, immunocytes and matrices to achieve growth, differentiation and repair, mainly via the collective actions of hormones/growth factors, i.e., PTH, vit-D, steroids and calcitonin, as well as critical cytokines (e.g., M-CSF, RANKL, VEGF, and IL-6 family) [5,6]. Mounting evidence has clearly indicated that OCs play an integral role in bone remodeling vs. sparing osteoporotic conditions and diseases [7,8,9,10], and their progenitor(s) have been shown to derive from the Mo/MQ lineages in the presence of macrophage colony-stimulating factor (M-CSF) and receptor activator of NF-κB ligand/(RANKL) molecules [11,12,13,14]. OCs typically have a life span of ~2–7 weeks in vivo; yet, upon activation, they become giant cells with multinucleation (≥2–3 nuclei), thereby expressing tartrate-resistant acid phosphatase (TRAP), calcitonin receptor (CT-R), cathepsin-K, intergrins-α_v_β_3_ and specific MMPs, phenotypically capable of dissolving bone matrix [1,4,6,7,8,9,10,11,12,13,14]. Despite the key molecules, such as CD11b, F4/80, Ly-6C, c-Fms/M-CSFR, c-kit/CD117 and RANK, they have aided in identifying OC precursors (OCps) in mice and humans systems [7,11,15,16,17,18]. Their characterization continues to be challenging, mainly due to the mixed heterogeneity and ontogeny issues related to Mo/MQ precursor lineages, and the controversial results obtained [7,8,9,12,13,14].

Recent studies have revealed that DCs, Mo/Mϕ and OCs could share common progenitors [19,20,21,22,23,24,25,26,27]. For example, inflammation may trigger Mo progenitors to become either DC or Mϕ [8,21,22,25,26,27], whereas TNF-α is capable of skewing the Mo-Mϕ lineage to DCs in local tissue environments via M-CSF/R up-regulation and expression in the circulating OCp pools [13,24,28,29]. Further, Rivollier & Servet-Delpart demonstrated that murine BM-derived and human Mo-derived Flt3^+^ DCs can transdifferentiate into active OCs in response to M-CSF and RANKL in vitro and ex vivo, suggesting that DCs may act like OCp [19,21]. Myeloid DC-derived OCs, not matured or plasmacytoid DCs (i.e., CD11c^−^B220^+^DC), can manifest unique phenotypic features of OCs during osteo-endocrine/immune interactions with ample environmental cues [19,20,21,22,24,25,27,30].

DCs (i.e., immature myeloid DCs/mDCs) can engage T cells directly and form focal aggregates [31,32] through a critical RANKL/RANK-OPG triad via a TRAF-6/adaptor complex that prompts regulatory vs. pathogenic sequelae [23,31], suggesting their involvement with inflammatory bone loss in vivo [20,21,22,23,32,33]. Yet, how and why the multiple lineages (i.e., DC-Mo/Mϕ-OC cells, etc.) interact during the pathogenesis awaits further study. Consequentially, such interactions may have potential implications, providing clinical and/or therapeutic applications not only for modulating tissue inflammation and destruction but also for osteoclastogenesis leading to bone loss vs. formation or remodeling involved with tissue repair vs. regeneration in specific bony environments [1,10,20,25,28,33,34]. Herein, the present narrative review touches on the collective advancements with innovative findings focused on mDCs and their overall routes leading to osteoclastogenic pathways for their prospective impacts.

## 2. Myeloid Dendritic Cells (mDCs) Acting as the OCp In Vivo

### 2.1. CD11c^+^-Myeloid Dendritic Cells (mDCs) as OCps Heading to Osteoclastogenesis

Recent studies evidently showed and suggested that DCs of myeloid lineage/progenitors can act like OCps to mediate bone resorption in vitro and in vivo [17,19,20,21,22,25,26,27,35,36,37,38,39]. In parallel, one of the most direct and recent pieces of evidence that mDCs are indeed OCps in vivo derived from a set of serial gene target experiments by Puchner et al., where CD11c-diphtheria toxin/DT-receptor mice (CD11c-DTR) were treated with DT via serum transfer for arthritis (STA) and hTNF-transgenic arthritis mice (hTNF-tg) via clinical histo-morphometry, and those OCs/OCps were analyzed in CD11c-vs. Zbtb46-tomato “fate-reporter” mouse models using fluorescent imaging in vivo [40]. Their results unequivocally support the notions that (i) myeloid DCs substantially contribute to the genesis of OCs in mice and humans, and (ii) CD11c is the general marker defining all OCs under homeostasis and inflammation, in which (a) depleting CD11c^+^ cells in inflammatory arthritis models (i.e., STA vs. hTNFtg) in vivo ameliorates their cardinal features by reducing inflammatory bone destruction and OC production; (b) activated OCs do come from conventional DCs/cDCs, as all OCs in the “fate-reporter” mice are Zbtb46-Tomato^+^-positive cells (having been multinucleated TRAP^+^-OCs in the tibial bones); (c) hCD1^+^-expressing cDCs (i.e., CD14^−^CD1c^+^CD19^−^ or CD14^+^CD1c^−^CD19^−^) can and have readily differentiated into bone-resorbing OCs in vivo [40], consistent with the results of using whole genome-wide expression analyses, where mDCs, as OCps, that are enabled to act like bone-resorbing cells behave much more efficiently than classical Mo-derived OCs/OCps [41], possibly via programmed genetic/transcriptional profiling or epigenetic modification [41,42,43,44,45].

Such mDCs differentiate into an OCp subset and then become mature active OCs under certain conditions or specific factors, e.g., in the presence of nutrients in local microenvironments, as described previously [8,10,19,20,21,22,25,26,27,35,36,37,46,47,48,49]. These are termed as DDOCs or mDDOCps [20,22,25,27,36,39,50,51,52], and their underlying interactions have been considered to operate at different levels under homeostatic and/or pathogenic conditions [8,20,25,33,35,45,48,50], likely involving [50] (i) M-CSF and RANKL signaling upon lineage progression to OCp/OCs; (ii) a plethora of diverse T-cell subsets (i.e., Th1, Th2, Treg and Th17) for effector activities; (iii) DC-derived specific osteotropic vs. anti-osteoclastogenic cytokines (i.e., IL-23, IL-34, IFN-λ1, etc.), thereby promoting de novo (anti-)osteoclastogenesis [50,51]; and (iv) unique crosstalk(s) associated with some or any of the interactions addressed above [40,42,45,50,52]. After all, given the complexity of dynamic interactions between the host’s immunity and each of the unique local micro-environments at the osteo-immune/mucosae–mesenchymal interfaces, the following endeavors to decipher the underlying chain of events that (im-)balances the homeostatic vs. pathogenic outcomes applicable for clinical interventions will be very exciting in the coming years.

### 2.2. Contribution of TRAF6-Independent Signaling to mDC-Associated Osteoclastogenesis In Vivo

Interestingly, it has been shown that there are likely robust mDC-derived multinucleated TRAP^+^ OC cells in the local osteo-immune interface between the inflamed synovial and bone surfaces in the host’s local tissues, for example, more localized CX3CR1^+^OCp than CX3CR1^+^-Mϕ/OCp cells [40,45,48,50,51,53,54], highlighting their pivotal and critical role in (re-)shaping or (re-)directing the ultimate outcomes pertinent to homeostasis or pathology. Further, a recent study used single-cell RNA-sequencing protocols to analyze the fate of cells transiting from BM-derived precursors to mature OCs [i.e., monocytic or common myeloid progenitors (CMPs) developing into mature OCs], and discovered that they went through CD11c^+^-(ITGAX)^+^DC pools, whereas RANK depletion in such CD11c^+^ subsets drastically suppressed OCp/OC development, strongly suggesting that CD11c^+^DC-like precursors were primed and destined for OCp/OC differentiation and effector activity [55]. Though CD11c^+^ myeloid DCs (mDCs) may not necessarily be delineated exclusively to DCs and/or Mo/MQ lineages [7,24], some other studies strongly favored their direct correlations in vivo [47,55,56]. The traceable contaminant(s) from the minor Mo lineage of multinucleated DC-like OCp cells may exist or co-localize; notably, we have previously reported that [22,25,36,37] (i) only the committed mDCs showed CD11c^+^ expression in a BM-derived DC subset according to multiple limiting-dilution analyses; (ii) Mo/MQ depletion, via the gene-knockout/deficiency methods, did not deviate from mDC-associated OCp development and function; (iii) almost all post-activation committed mDC cells showed a CD11c^+^CD11b^−^TRAP^+^-multinucleated OC phenotype according to FACS analyses, thereby confirming the CD11c^+^OCp status described above [22,25,36,37]. Likewise, other definitive reports also support that a separate subset of CD11c^+^Mo-lineage cells with DC-like phenotypes may develop into classical or conventional DCs/cDCs upon transmigration through the endothelium [47,57], whereas CD11b expression becomes down-regulated, as OCps develop into active OCs [28,42,56,58]. Therefore, such minor traces of contaminant(s) are highly unlikely to exert a significant role in their development into CD11c^+^TRAP^+^DC-like OCs associated with inflammatory bone loss in the models analyzed [47,52,56,57].

Moreover, the same notion described above was further reconciled in a recent study [52], where the influence of CD11c^+^-mDCs, as OCp/mDDOCps, on inflammation-induced osteoclastogenesis ([14,25,33,35,36,39]; via chicken type-II collagen/CC-II challenge) was examined in lethally irradiated TRAF-6^(−/−)^-null BM chimeras (thus termed T6KO_bmChi) without the endogenous presence or involvement of classical so-called Mo/Mϕ-derived OCp cells in vivo [59,60]. As a result, there were more quantifiably dually labeled CD11c^+^TRAP^+^DC-like OCs detected in the focal bony surfaces and inflamed joints of CC-II-immunized T6KO_bmChi mice in vivo, representing the majority and most accountable local/residential bone-resorbing cells, more so than those singly labeled TRAP^(+)^CD11c^(−)^ cells (i.e., CD11c^(−)^Mo/Mϕ-derived TRAP^+^OCs) in arthritic tissues/joints as detected and measured in CC-II-immunized WT_bmChi controls [52]. Such featured findings can be extended to the DBA mouse strain, a highly responsive CC-II-induced inflammatory/autoimmune arthritis model [25,36,39]. These observations are in high accordance with the new paradigm reported above [40] that myeloid CD11c^+^-mDCs are substantially attributed to the primed CD11c^+^TRAP^+^TRAF6^(−/−)^DC-like migratory OCs and selectively located in the inflamed synovium vs. bone surfaces in TRAF-6^(−/−)^-null chimeric mice [52], contributing to arthritic bone loss without the influence of Mo/Mϕ-derived OCp, challenging prior thoughts on discovering or defining the roles of classical Mo/Mϕ-derived OCs [1,11,12,13,14,16,28,40,41,45,50,58].

To this end, immature CD11c^+^mDCs are clearly involved in engaging osteo-immune interactions that lead to de novo alternative route(s) into robust osteoclastogenesis without the classical Mo/Mϕ-derived OCp/OC cells in vivo. Prospectively, further studies employing genetic tags with molecular beacons, e.g., [40] parallel cell-kinetics analyses of the designated progenitors of mDC/OCp lineage(s) from upstream ontogenies through the time course in vivo, will be very interesting, enabling us to address and delineate the subsequent contributions and/or attributable impacts of their downstream lineage(s) until they become (non-)myeloid-featured multinucleated CD11c^+^TRAP^+^DCs/OCs, which are one progeny of CD11c^+^mDCs/OCp cells, during development vs. differential contributions to the subsequent and/or parallel concurring pathways of osteoclastogenesis and the overall bone loss encountered as well, by comparing them to classical Mo/Mϕ-derived OCs for further insights.

### 2.3. Cytokine Crosstalk as Unique Signals to Modulate mDC-Associated Osteoclastogenesis In Vivo

Though TRAF-6-independent osteoclastogenesis has previously been reported via the gene-knockout approach [59,60], along with the recent findings described above (see Section 2.1 and Section 2.2 and refs. [40,45,50,52]), such ontogenic developments, lineage interactions, and contributions to subsequent progression pertinent to bone loss vs. genesis/repair or remodeling remain elusive and not totally clear at present. Intriguingly, TGF_β_-vs.-IL-17 cytokines’ crosstalk is involved in modulating a unique alternative osteoclastogenic route through systemic administration of cytokines and/or anti-cytokines blocking mAbs in vivo [52], in essence supporting the notion that the development and interactions of mDCs/mDDOCp subsets related to arthritic bone loss signify distinctive step-wise events, with or without the TRAF-6/adaptor signaling among the Mo/Mϕ-derived OCp pools, within the local microenvironment that predisposes to pathogenic progression under inflammation [39,40,52,61,62].

**Figure 1:**
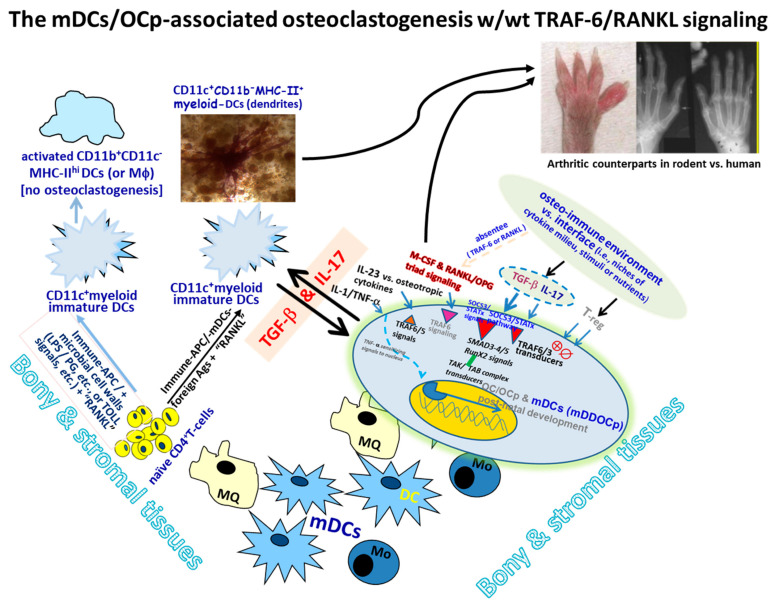
Myeloid dendritic cell (mDC)-associated osteoclastogenic pathways are developed with or without TRAF-6 or RANKL signaling, as depicted. Herein, it is shown that the cytokine TGF-β remains critical to priming CD11c^+^mDDOC/mDDOCp cells deficient in TRAF6-related immune/osteotropic signals, featuring TGF-β- and IL-17-invoked effectors in the environmental milieu sufficient to drive bona fide osteoclastogenesis in vitro/in vivo [25,36,40,50,52]. Clearly, myeloid CD11c^+^DCs/mDCs (immature cells are lighter, mDCs are darker blue) can contribute directly to osteoclastogeneic pathways through the TRAF-6-signaled classical Mo/Mϕ subsets involved (i.e., the lighter yellow cells), without any contribution of such classical Mo/Mϕ subsets even in the absence of TRAF-6-mediated signaling in vitro/in vivo (i.e., the pinkish highlights in arrows and boxes, and refs. [25,27,36,40,50,52,59,60]). In parallel, multiple environmental and/or in situ factors can modulate and engage to trigger such step-wise unique osteo-immune crosstalk(s) as described in the manuscript text. Moreover, there are plausible additional intertwined players (IL-23, TNF-α, IL-1, etc.), other than the known Th-effectors and Treg cells, with previously reported intricate interactions (i.e., the green-colored signaling events in the CD11c^+^mDDOC/mDDOCp cells, and refs. [35,50,52]) to fully circumvent TRAF-6^(−/−)^-null signaling cascades sought traditionally, unless TGF-β or Mo/Mϕ-derived-OCp or mDDOCp-like cells are readily available in the paracrine or juxta-paracrine fashion in the local environments.

Therefore, it is clear that the immature myeloid-CD11c^+^TRAF6^(−/−)^DCs (or mDDOCp) manifesting the precursor phenotype, acting as OCps, can promptly develop into active and functional OCp/OCs for osteoclastogenesis in the absence of endogenous Mo/Mϕ-derived classical OCp/OCs in vivo, whereas TGF-β-mediated step-wise regulation of the de novo mDCs/OCp, through a distinct crosstalk with IL-17, provides unrecognized molecular interactions featuring TRAF6^(−/−)^ CD11c^+^mDCs/OCp readily present in the local environment, driving a bona fide alternative route of inflammation-induced bone loss (see Figure 1 below and refs. [40,50,52]).

**Figure 2:**
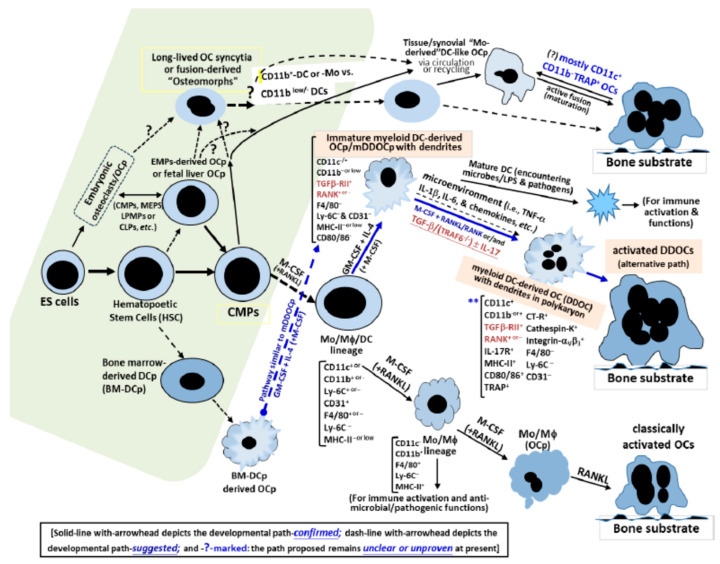
The development of osteoclastogenic pathways: bona fide mDC/mDDOCp(OCp)-associ-ated osteo-immune interactions for inflammation-induced bone loss revisited. The immature CD11c+ myeloid dendritic cell (mDC)-associated route is developmentally involved in alternative vs. classical bona fide osteoclastogenesis. The immature CD11c+myeloid-mDCs/mDDOCp/OCp-mediated osteo-immune interactions are associated with alternative route as part of the confirmed (e.g., proven link by the solid-line), suggested or unclear/unproven links (e.g., dash-line with or without question mark-?- in pathways)for inflammation-induced osteoclastogenesis through the hematopoietic developments leading to the pathogenic vs. homeostatic bone loss and/or remodeling processes, respectively, as depicted. **Note: (i)** The solid-line with arrowhead depicts the developmental path-confirmed; dash-line with-arrowhead depicts the developmental path-suggested; and **-?-**marked: the path proposed remains unclear or unproven at present. (i.e., see the green-shaded areas); **(ii) Abbreviations:** ES: embryonic stem cells; HSC: hematopoietic stem cells; BM: bone marrow; EMPs: erythro-myeloid progenitors; CMPs: common myeloid progenitors; MEPS: megakaryocyte-erythrocyte progenitors; LPMPs or CLPs: lymphoid-primed multipotent progenitor or common lymphoid progenitors, respectively; mDDOCp: immature myeloid DC-derived OCp; Mo/Mφ: monocytes & macrophages; DC: dendritic cells.

Nevertheless, the above studies have advanced our understanding of the bioactivities of specific OCp/OC subsets or lineages. Herein, we summarize the central findings relevant to the osteoclastogenic pathways involving ontogenic developments and the classical vs. alternative routes leading to subsequent bone loss, as shown in the proposed schematic illustrations, mimicking a roadmap that highlights the upstream paths derived from the plausible ontogenies reported and the consequential downstream paths. The evidence is graphically simplified in the scaled solid and dashed lines depicted in Figure 1 and Figure 2 below.

## 3. Ontogenic Lineages vs. Collateral Routes Associated with Osteoclastogenic Developments

Prior studies have suggested that DC-lineage differentiation may start close to the HSC [63], at which the transcription factor interferon regulatory factor-8/(IRF8) regulates the organized chromatins at the lymphoid-primed multipotent progenitor (LMPP) stage, or the common lymphoid progenitors (CLPs), to induce early commitment(s) toward DC lineage development [43]. Thus, whether some OCps or OCp subsets can be derived through some alternative route(s), such as LMPP or CMP-derived DCs, remains to be studied to determine when and how such DC-OCp/OCs lineage specifications or conversions are initiated and delineated during development vs. how the stage-specific fates are determined sequentially.

An earlier study using an induction stimulus and the reciprocal rescue approach (e.g., transplantation protocols) with cell suspensions prepared from the spleens and BM of osteoporotic hosts indicated the hematopoietic origins of OCs/OCps [64,65]. Moreover, recent studies analyzing the ontogenic lineages of OCp emergence have strongly supported that the differential and continuous waves of seeding erythro-myeloid progenitors (EMPs) produce the embryonic/yolk-sac Mϕ and tissue-residential Mo (i.e., in brain and fetal liver) at E7–7.5 (embryonic OCs), followed by the emergence of fetal HSCs in the vasculature of the BM cavity at E8.25–9 till around E10.5–15.5, giving rise to hematopoietic OCp pools [66,67,68,69].

It is now clearer that both EMP-derived Mϕ and HSC-derived Mo can produce “embryonic” and “post-natal” OCp cells, hereby termed “fetal Mo-derived OCp” pools [70,71], rendering a rather complex network of multiple developmental origins, based on recent novel findings [72,73]. In other words, it is evident that multinucleated TRAP^+^OCs can be observed as early at E16.5 in mouse embryos whose HSC-derived OC progenitors (OCps) have been removed, indicating that (i) primary OCs arising during early osteogenesis without hematopoietic seeding are derived from EMPs, rather than fetal HSCs [70]; (ii) EMP-derived OCs/OCps play an important role in the generation of BM niches or cavities, thereby promoting the migration of HSCs and myeloid progenitor cells (i.e., CMP [45]); (iii) embryonic EMP-derived OCps and fetal HSC-derived Mo-progenitors/CMPs, including CX_3_CR1^+^ and CSF1R^+^ yolk-sac Mϕ, respectively [45,74], as well as post-natal BM/HSC-derived OCps (e.g., yielding Mo/Mϕ and DCs subsets) through various differentiation processes, can fuse to form notably complex yet diverse OCp/OC (sub-)populations [45,70,71,72,73].

Of interest, EMP-derived OCp cells are gradually replaced by HSC-derived CMP cells [45], yet certain Mo progenitors have been reported that can fuse with the “long-lived” EMP-derived OCs, thus maintaining their populational levels or pools throughout adulthood [70]. However, these multi-faceted developmental vs. consequential connections among the EMP-derived vs. HSC-derived OCp cells in the timely staged vs. spaced (sub-)populations and the immature CD11c^+^mDC precursors (e.g., abundant in bone–mesenchyme/mucosal interface and tissues) remain largely unclear and are still in their infancy, awaiting further studies (see the green-shaded areas of Figure 2 and refs. [45,73]).

## 4. The Turn-Around and Beyond vs. Turn-Over of OCp Cells and Mature OCs

Traditionally, OCs are thought to last on average about ~2 weeks, enabling them to complete their active bone resorptive activities before undergoing apoptosis [6,9,10,13,14,74]. However, a recent study has suggested that individual OCs can be rather long-lived instead, acquiring a renewed or new nucleus every 4–8 weeks from the circulating HSC-derived blood cells; hence, there is a 6-month period to renewing ≥5 nuclei (ranging from 3 to 7) at the individual OC level [70]. In addition, these OCs are capable of surviving much longer by fusing with circulating Mo cells with access to remodeling units [45,70], so the focal bone remodeling progresses within the canopy, which has conceptually challenged this traditional view [75]. Meanwhile, the “intravital-imaging” technique has also unexpectedly revealed the cell fate of mature OCs, which can be divided into some motile smaller OC-like “un-resorbing” cells, termed “osteomorphs” [76,77], via fusing with neighboring OCs or in some cases with each other, and then are “re-cycled further” into bone-resorbing OCs under RANKL-controlled stimulation, where they become highly motile OC daughter-cell pools in the adjacent BM and retain the ability to fuse back to become mature OCs, a reversible process during the dynamic OC-OB interactions associated with physiological homeostasis or repair in the bone-remodeling units [76,77].

As for the mDCs and OCp pools described (i.e., behaving at the intermediate stage or as transient OCs, i.e., osteomorphs), it may be rational to theorize and consider that such fusion of OCs in multinucleation processes may likely be non-random, but modulated through discrete mechanism(s). Thus, such fusion partners are selected or screened based on the heterogeneity of OCs and OCp cells in the local environment or in some special conditions (i.e., reverse RANKL signaling [78]), where osteocytes and stromal or OB cells are in proximity within the bone matrices. Therefore, the progeny from their ontogenic lineages is probably determined at the earlier stages of hematopoiesis while adjusting to operational and physical requirements during the different phases of bone-remodeling or -repairing conditions. Once they have been well-studied and revealed in full mechanistic detail, further clinical implications could be directly relevant and subject to practical applications for osteoporotic conditions or therapy in the future.

Nevertheless, any probable or plausible alternative route(s) engaged may be implicated and involved with, for instance, either influx(es) of the precursors’ egress from local circulations or recruitment of early mDCs/OCp pools in situ in (pro-)inflammatory conditions in response to local calls or stimuli, including juxta-crine and hormonal signals [79,80], chemotactic signals [9,30,57,73,81,82,83] or mechanical cues [84]. An emerging concept of the heterogenous lineages of OCs’ development, bio-functions and fates has been proposed [45,67,73,76,85]; in principle, their potential interactions may be intertwined with traces of the co-localized granulocyte-Mo progenitors (GMPs), EMPs or Mo-OC-DC progenitors (i.e., MODP, CD11b^−^CD31^+/hi^ Ly6C^−/+^C-kit^+2^), Mo-derived CD11c^(+)^DCs, plasmacytoid DCs of GMP lineage, or bona fide CD11c^(+)^ TRAP^(−)^DCs in situ, which have been timely silenced or less adapted [like ~TRAP^(−)^] to embrace the typical OC-like phenotype(s) under inflammation in vivo [12,24,28,52,60,72]. Additionally, EMP-derived primary OCp cells could migrate from the spleen to the injured sites of fracture through the bloodstream [71], though the mechanism of splenic cell recruitment to the fracture site(s) remains unclear. Notably, it has been shown that OCp subsets can be space–temporally divided into certain sub-types based on the levels of cell mobility and resorptive profiles through their post-activation status and/or fracture repair [48,73]. Moreover, increasing evidence has also suggested that though BM cells are the major source of OCps during tissue-dynamic homeostasis, some of the circulating Mo cells may likely be a part of that source for OCp/OCs in pathogenic conditions, such as fracture repair, poly-inflammatory/autoimmune diseases, etc. [48,86].

## 5. Discussion

At present, it is sufficiently clear that during development, early embryonic EMPs can generate “primary OCp” pools for subsequent hematopoiesis and/or fracture repair, whereas HSCs maintain the capacity for self-renewal and proliferation, yielding the multi-potent progenitors and thereby producing specific lineages of CMPs, MEPs, LMPPs, CLPs, etc. [48,66,67,68,69,70,71,72,73,85,86,87]. Yet, recent ample evidence appears to have indicated that (i) primitive EMPs, CMPs, MEPs, CLPs, etc., may enable to generate some early OCp pools and/or the long-lived kinds [6,7,9,12,48,55,66,67,68,69,70,71,72,73], either before or after entering the local tissue niches and blood circuits upon activation [10,25,26,27,28,35,52] or recruitment (i.e., sphingosine-1-phosphate and stroma-derived factor-1; [49,88]); (ii) all of these OCp subsets may develop into mature fused OCs via M-CSF and RANKL signaling; and (iii) locally dwelling Mo and tissue-specific Mϕ cells can be triggered to become active OCs directly [6,7,8,9,10,11,12,13,14,20,21,22,23,25,26,27,28,29,36,55,77,85].

Further, these recent findings could coherently suggest that through the upstream ontogeny (i.e., embryonic vs. HSC-derived OCp pools), collaterally intermediate post-(ES/HSC)-natal steps in the BM or juxta-BM stroma/circulations (i.e., EMPs, CMPs, MEPS, LPMPs, CLPs, BM-DCp, etc.) and the routes preceding the junctional intermediators in the regional tissues and blood circuits downstream (i.e., some of BM-derived Mo/Mϕ/DC precursors, MODP, mDDOCp, etc.), there are likely multiple OC/OCp-type loops on their paths, forming a continuous and connected network for osteoclastogenesis (see Figure 2), subject to the proper modulations throughout various developmental stages of timing, lineages, tissue specifications at the check-points, cell types engaged and/or fine-tuned, local niches, and blood circulations, leading to the regulatory vs. pathogenic sequelae. To this end, it is paramount to explore and address, dependently or independently, their complex interactions with the traditional so-called Mo/Mϕ-derived OCp/OCs subsets analyzed, studied and modeled for the coming years.

Moreover, it is prudent to address that there is new evidence suggesting that the underlying molecular interactions, with or without TRAF-6-transduced signaling cascades, in CD11c^+^mDCs cells and other comparable OCp subsets associated with the classical and/or alternative routes for osteoclastogenesis are rather complex, along with different paths in play around the environmental cues leading to the consequential bone resorption vs. remodeling. Nevertheless, those recent novel findings and the present perspectives are in good concordance with other new scholarly reports (see Figure 1 and Figure 2), where the mCD11c^+^DCs/mDDOCp manifests the precursor phenotype, as OCps can develop into active and functional OCs for osteoclastogenesis, despite the absence of endogenous Mo/Mϕ-derived classical OCps in vivo, where the step-wise TGF-β-mediated regulations of de novo immature mDCs/OCp cells, through some distinct crosstalk(s) with IL-17, provides an innovative insight into the underpinning framework featuring TRAF6^(−/−)^CD11c^+^mDDOCp readily present in the local microenvironment to compatibly drive a bona fide alternative route towards inflammation-induced bone loss.

Intriguingly, such timing along with sequential kinetic responses of immature CD11c^+^mDCs/DC-derived OCp/mDDOCp have been shown to be in much swifter and faster dynamics than those of the classical Mo/Mϕ-derived OCp subsets, upon transcending the signals (i.e., either active OCs or primed APCs) for orderly modulating bone resorption vs. remodeling, based on the phenotypic features revealed [22,25,26,27,28,29,30,31,32,33,34,35,36,52], or even including the potential applications leaning towards bone repair and/or regeneration downstream into the future aspects [89].

## 6. Future Perspectives

Paradoxically, some exemplary areas worthy of exploration to advance the field for better insights and understanding into potential implications for physiologic applications include (i) the space–temporal kinetics of the primitive/hematopoietic OCp pools during development vs. downstream differentiation and effectors, i.e., specific interactions with OCp-OB/stromal cells or similar for the coupling vs. de-coupling of osteoclastogenesis or osteogenesis related to growth, remodeling, regeneration, or repair; (ii) the roles/contributions of hematopoietic progenitors vs. mesenchymal stromal cells underlying the primitive osteoclastogenic pathway(s) associated with “embryonic vs. post-natal” OCp cells leading to differential homeostatic vs. pathogenic outcomes; (iii) the molecular determinants of the transiently mobile further-recycled osteomorphs for the gain vs. loss of function in remodeling units during OCp-OB/stromal crosstalk/interplays (i.e., mDCs, cytokines, etc.) leading to classical and/or alternative osteoclastogenesis, where each of which will likely give rise to meaningful impacts on the prospective clinical applications in osteology and osteo-immunology research.

## 7. Conclusions

Collectively, it is now clearer to suggest that there are likely complex networks for the pathways of osteoclastogenesis at different stages of the host development, from the ontogenic primitive OCp pooled lineages to the later post-natal OCp subsets intertwined with some specific cell-to-cell interactions proxy-positioned via the regional osteo-immune/mesenchymal interface and/or collateral tissue/residential sites leading to the establishment of osteoclastogenic routes (see Section 3), likely involving the focal bone-remodeling units localized in the periphery (i.e., with motile recycled osteomorphs, etc.; see Section 4). Overall, such complex osteo-immune interactions determine classical vs. alternative osteoclastogenesis as depicted in Figure 1 and Figure 2, from the upstream ontogeny down to the subsequent paths through the continuous and connected multiple OCp/OC-type lineages in the networks illustrated, and will require more studies of their molecular interactions to decipher their physiologic sequelae and impacts via some useful in vivo models to analyze their human counterparts, including the inflammatory pathogenesis of arthritis, osteoporotic disorders, periodontitis, etc., in the future.

## Data Availability

The authors declare that the drawings or data supporting the present prospective article are available from the authors upon reasonable request.

## Abbreviations

The following abbreviations were used in this manuscript, in alphabetic order:

CC-II	Chicken type-II collagen
CMP	Common myeloid progenitors
cDCs	Conventional dendritic cells
CT-R	Calcitonin receptor
DCs	Dendritic cells
DDOC	Dendritic cell-derived osteoclast
DTR	Diphtheria toxin receptor
EMP	Erythro-myeloid progenitors
GMPs	Granulocyte-Mo progenitors
HSC	Hematopoietic stem cell
hTNF-tg	hTNF-transgenic arthritis mice
mDCs	Immature myeloid DCs
IRF8	Interferon regulatory factor-8
LMPP	Lymphoid-primed multipotent progenitor
M-CSF	Macrophage-colony stimulating factor
MMPs	Matrix metalloproteinases
Mo/Mϕ	Monocytes/macrophages
MODP	Mo-OC-DC progenitors
mDCs	Myeloid dendritic cells
mDDOCp	Myeloid DC-derived OCp
OBs	Osteoblasts
OCs	Osteoclasts
OCp	Osteoclast precursor
PTH	Parathyroid hormone
TRAP	Tartrate-resistant acid phosphatase
TRAF-6	Tumor necrosis-associated factor-6
T6KO_bmChi	TRAF-6(−/−)-null bone marrow chimeras
RANK	Receptor activator of NF-κB
RANKL	Receptor activator of NF-κB ligand
STA	Serum transfer for arthritis
VEGF	Vascular endothelium growth factor
